# Fresh air in the city: the impact of air pollution on the pricing of real estate

**DOI:** 10.1007/s11356-023-31668-1

**Published:** 2024-01-02

**Authors:** Oktawia Miłuch, Katarzyna Kopczewska

**Affiliations:** Faculty of Economic Sciences, University of Warsaw, Ul. Dluga 44/50, 00-241 Warsaw, Poland

**Keywords:** Air pollution, Spatial econometrics, Real estate valuation, Q53, R31, C21, O18

## Abstract

This study investigates the impact of ambient air pollution on housing prices in Warsaw, Poland, by examining spatial dependencies. The high concentration of particulate matter (PM_10_, PM_2.5_ and PM_1_) is expected to reduce real estate values. Using a hedonic model with approximately 15,000 observations and a spatial error model, we did not find evidence of this impact. Standard and premium housing submarkets differ in price determinants, but both are insensitive to environmental issues. This could be explained by the lack of comprehensive intra-urban historical information on air pollution, which limits investors’ rationality and their ability to properly value real estate based on environmental issues. Additionally, measurement and aggregation issues, along with low pollution variability within the city, may contribute to the insignificance of this information in real estate sales prices. Our empirical research confirms a strong link between air pollution and weather conditions within the city, where low temperatures and low-speed southern winds worsen contamination levels, while high temperatures and westerly winds improve air quality. Furthermore, we find that incorporating pollution data using PM yearly mean concentration works better in modelling than the PCA-reduced air pollution index.

## Introduction

Despite the extensive research on air quality, the real estate value created by clean air in an urban environment is still unknown as the empirical studies are far from conclusive. There are three reasons for this.

The first one, theoretical, lies in *rationality assumption* and the interaction of many factors impacting housing prices. Environmental characteristics are not market goods; thus to estimate the value of air quality, the most common approach in economic studies is to link it with real estate value from a given area. In a pioneering work, Ridker and Henning (1967) assumed that air quality was one of the variables explaining a potential home buyer’s utility function. With the hedonic price theory development, the air pollution assessment was mainly based on housing prices. They assumed that when controlling structural attributes, the observed differences in real estate prices exhibit the site characteristics value, such as environmental amenities (see meta-analysis by Smith and Huang 1995). However, such a perspective implicitly assumes that investors^1^ can consciously choose (and valuate) at a given moment in time all of the features of real estate. This is mostly true in relation to the size, location, or interior features such as balcony, etc., but difficult in the case of air pollution. This is because the current measurements of air quality are usually well known, but the long-term historical and prospective information about the environmental conditions of a given location is often unavailable to investors. There is also due to temporal variability of air quality — air pollution changes over seasons with weather conditions (temperature, wind). Investors can average this phenomenon or take status on the day (e.g. exceptionally good or bad) — their approach is unknown when using transaction data. A lack of long-term environmental information or lack of choice in the real estate market (higher demand than an available stock of flats) limits the possibility of making rational decisions and thoroughly evaluating this factor.

The second reason lies in the *methodological approach*. A literature review reveals a few methodological aspects that should be particularly taken into account. One is the *spatial aspect of the analysis*. As pollution and housing prices are similar in the neighbourhood, one must consider spatial autocorrelation. In the early studies, the spatial dimension of data was often neglected (Ridker and Henning 1967; Smith and Huang 1995), which usually led to biased results. However, with a paradigm shift towards spatial econometrics modelling, the practice has changed (e.g. Anselin and Lozano-Gracia 2008; Fernández-Avilés et al. 2012; Lu and Lee 2022). Another one is the *choice of pollutants* included in the research. There are two common streams: to examine the influence of the most perceptible air contaminants, such as PM_10_ or ozone (Chay and Greenstone 2005; Anselin and Le Gallo 2006; Kim and Yoon 2019), or to use a synthetic measure (often the weighted average of different pollutants), such as the air pollution index (e.g. Anselin and Lozano-Gracia 2008; Fernández-Avilés et al. 2012; Lu and Lee 2022). We show there are advantages and disadvantages of both. However, the point is a variety of information — very few studies have examined the impact of PM_1_ or considered jointly all types of particular matters, which pollution measurement stations typically report. The inclusion of PM_1_ data in the annual cross-section is a novelty and contributes to the literature.

Finally, there is a *mismatch between monitoring station locations and residential sites*. Most papers use only a few air monitoring stations and have to apply interpolation techniques, such as ordinary kriging, to obtain the value of environmental variables in the house location. However, as stated in Anselin and Lozano-Gracia (2008), the usage of interpolated environmental variables leads to the prediction error correlated with the overall pricing model error, which may result in a bias in the estimation. There are two possible solutions to that. One is to use an endogeneity toolbox, e.g. instrumental variables and the two-stage least squares (2SLS) method when estimating the model (Anselin and Lozano-Gracia 2008; Lu and Lee 2022). However, the results are ambiguous. The second one is to use densely located air monitoring stations — usage of real spatially close data is computationally less complex and seems much more suitable and precise as it does not use any interpolated data and thus is free from bias resulting from the spatial distribution of monitoring stations and housing locations.

In this study, we hypothesise that the local air quality in the urban environment has a very moderate impact on real estate pricing. This is due to a lack of the investor’s control over this factor and the imperfect knowledge of long-term environmental conditions in a given location. Moreover, we claim that moderate air quality variation within the city causes air quality to be very similar in different districts; thus, it is not a differentiating factor. Also, we investigate if proper spatial techniques, together with spatially precise data and detailed pollutants measurements, can improve the quality of analysis. This research aims to examine the influence of air quality on real estate valuation in Warsaw, the capital city of Poland. It is an important metropolis in Central-Eastern Europe, but poorly examined in this context. The remainder of the paper is as follows. The literature review in the “Literature review” section analyses models, data and methods used in studies on air quality’s impact on housing prices. The “The empirical study of air quality in Warsaw” section presents environmental data analysis and a methodological discussion on air quality assessment. The “Analysis of housing market data in Warsaw” section reports the housing data, while the spatial hedonic model, including air quality, is discussed in the “The hedonic model with air quality — methodology and results” section. The paper ends with policy implications and conclusions.

### Literature review

There are at least three essential aspects to consider in the relationship between air quality and housing valuation: (i) rationality in housing valuation due to pollution, (ii) data aggregation and its spatial dimensions, (iii) quantification of air pollution. We discuss them below.

#### Rationality in housing valuation due to pollution

Since the early studies on the value of clean air (Ridker and Henning 1967; Rosen 1974), the hedonic pricing model for housing data has long been the predominant methodology in environmental economics, even if conclusions are ambiguous. In a model proposed by Rosen (1974), a consumer good is a bundle of characteristics that determines its price. In the context of real estate, a flat’s features can be divided into three groups: structural attributes, the provision of neighbourhood services and environmental resources. In the hedonic price method, the characteristics of the given product impact the utility associated with the consumption of various goods — furthermore, the existence of diversified goods implies a wide variety of alternatives. Under the assumption of perfect competition and information, both consumers and producers make decisions that maximise their utility. For instance, according to the hedonic price theory, environmental disamenities, such as air pollution, should negatively impact the housing price with all other features constant. Based on that, numerous studies have been conducted and summarised in extensive reviews, among which are the meta-analyses presented by Smith and Huang (1995) and Chay and Greenstone (2005). The former study indicates a rise of 0.05–0.1% in real estate prices due to a reduction in the total suspended particulates by one unit (micrograms per cubic metre). In the latter paper, the conclusions are ambiguous. The authors emphasise the difficulty of obtaining consistent results while using the hedonic approach as there may be unobserved characteristics correlated with local air pollution. For example, more polluted areas tend to be urbanised and densely populated regions with higher *per capita* incomes and crime rates. In both studies, most of the reviewed papers were conducted on county-level measures from the USA (the problem of data aggregation will be discussed in the next section). Apart from the problem of omitted variables which may bias estimates, the weak results of the empirical studies may be directly related to assumptions of the hedonic theory. The hypothesis about maximising utility depends on the belief that rational consumers make decisions based on the available information. However, as Simon (1972) has stated, bounded rationality becomes substantial as the complete information about possible alternatives is realistically impossible for most decisions. Since it is rational to assume that air quality influences real estate prices, it is also reasonable to presume that not all consumers are aware of the long-term environmental characteristics of a property neighbourhood as, in many cases, such information is not publicly available. Therefore, consumers may not include the air quality of the neighbourhood when purchasing real estate.

#### Data aggregation and their spatial dimensions

The crucial technical aspect of many spatial analyses is the level of data aggregation and problems emerging due to data collection. In the pioneering paper of Ridker and Henning (1967), the authors based their study on observations by census tracts within a single metropolitan area (St. Louis) in 1960. The proposal to explain property value differences resulting from variation in air quality within locations contributed to the economists’ approach to measuring willingness to pay for environmental public goods. In the Chay and Greenstone (2005) meta-analysis, the authors emphasise the difficulty of obtaining consistent results while using the hedonic approach as there may be unobserved characteristics correlated with local air pollution. Therefore, a common approach is to apply environmental regulation as an instrumental variable. However, this might be inappropriate as air quality policies may adversely affect the labour market and impact negatively economic factors such as earnings or employment. In consequence, the housing market also might be negatively impacted as well as individuals’ willingness to pay for unpolluted air (Amini et al. 2022). While Chay and Greenstone (2005) took considerable care in tackling the issue of omitted variables, their study is performed on aggregated data from US counties. Their results indicate that a decrease of 1 µg/m^3^ in the TSP (total suspended particles) causes an increase in the housing price by 0.2 − 0.4%. Meta-analysis of 37 studies (Smith and Huang 1995) suggests that the unit change (µg/m^3^) of pollutants will result in a property value change ranging from 0.05 to 0.1% in the opposite direction. Most of the analysed works were based on data (to some extent aggregated) concerning metropolitan areas in the USA. A more up-to-date study conducted by Ou et al. (2022), using 13-year panel data from 237 prefecture-level Chinese cities, examined the premiums of clean air, measured by the concentration of PM_2.5_, capitalised in the real estate market. The results of their 2SLS hedonic model indicate a significant negative association between pollutants and housing prices. Namely, the results stipulate that a unit increase in PM_2.5_ level on average decreases the property value by 0.32%.

In recent years, the number of studies concerning Asia has increased considerably as air quality is one of their most severe environmental and economic concerns. Kim and Yoon (2019) studied the influence of air quality, using the average annual concentration of PM_10_ and the mean change of air quality in 2015 and 2016 on the housing prices in Seoul. Based on over 100,000 individual housing transactions, the authors applied the spatial SAC model to explain the natural logarithm of the dwelling’s price. As a result, the coefficient of PM_10_ turned out to be statistically insignificant; however, the change between 2015 and 2016 in air quality was significantly and negatively associated with property value. Another example of an Asian case study is the research conducted by Lu and Lee (2022) in South Korea. They propose an air pollution index (API) to assess the influence of air quality on the real estate market, following Anselin and Lozano-Gracia (2008) and Fernández-Avilés et al. 2012. Housing data concerning individual transactions from the whole territory of South Korea were collected between 2015 and 2018. Additionally, to capture spatial dependencies of various types, the 2SLS approach has also been introduced with instrumental variables (spatial coordinates and spatially lagged API). The results indicate that holding other factors constant, the 1% increase in air pollution led to a decline in property value by 0.32%.

Anselin and Lozano-Gracia (2008) applied an explicit spatial econometric approach, namely the spatial SAR model combined with 2SLS estimation, to a sample of individual house sales in California (four counties). Their results indicate a significant and negative impact of contaminations on property value. However, following a joint methodological approach, Fernández-Avilés et al. 2012 concluded oppositely. In the mentioned research, based on the point data from Madrid, Spain, the authors compared the maximum likelihood (ML) procedure and the spatial 2SLS method applied to ordinary least squares (OLS) and spatial Durbin (SDM) models. To implement the 2SLS, spatial coordinates and spatially lagged API were instrumental variables. What is more, irrespective of whether the API was considered exogenous or endogenous (with instrumental variable and without), and irrespectively of whether using spatial autocorrelation or not (SMD and OLS), there was no evidence that air pollution had any impact on real estate value.

To examine the relationship between housing prices and air quality, Tang and Niemeier (2021) used high-resolution mobile-based air pollution mapping data covering three major areas in Oakland, California. Considering housing and air pollution data, the spatial lag model (SAR) was constructed. The authors included an additional instrumental variable (mean of the median vehicle speed within the buffer area of 400 m) to address the endogeneity concerns. All pollutants included in the model were statistically significant and with a positive sign based on the estimated coefficients. Le Boennec and Salladarré (2017) examined the impact of air quality and noise on the real estate market in the metropolitan area of Nantes, France. Concentrations of pollutants were simulated using the Atmospheric Dispersion Modelling System (ADMS Urban model), a comprehensive system for modelling air quality. In the final model, the authors included the average concentration of nitrogen oxides. According to the paper’s conclusions, the most accurate method was the OLS approach which outperformed the spatial econometric models. Moreover, spatial autocorrelation between observations was insignificant. To summarise, the air pollution in Nantes had no impact on housing prices.

Anselin and Lozano-Gracia (2008) and Lu and Lee (2022) have indicated the statistically significant and negative impact of air pollution on housing prices, while other discussed studies do not confirm these conclusions. One of the possible explanations for this discrepancy is that the two former papers referred to comparatively large areas (respectively, a surface area of around 230,000 km^2^ and a country). Thus, the observed variability in the air quality is likely to be higher when concerning vast surfaces. Consequently, a potential homebuyer may be more sensible about pollution when the differences between locations are more tangible than within a city. This proposed explanation was also highlighted in Fernández-Avilés et al. 2012, and the results of the contemporary studies presented in the review appear to confirm it.

Considering the Polish real estate market, Ligus and Peternek (2017) analysed the value of a property’s environmental characteristics. Using four methods, linear, logarithmic, spatial error model (SEM), and a spatial autoregressive model (SAR) applied to the housing data concerning Warsaw, Poland, the authors estimated separate models for each city district. The environmental variables included in the research were noise, PM_10_ and nitrogen dioxide concentrations. In most districts, the mentioned characteristics were revealed to be statistically insignificant.

Overall, comparing the results of studies with respect to data aggregation and its spatial range (covered area — city, metropolitan area, country), one can find some regularities. Papers based on aggregated data tend to confirm the hedonic theory hypothesis about air quality impacting real estate value, even if the revealed dependency is weak. In the case of studies conducted with the use of point data, the results are ambiguous.

#### Air pollution, so what precisely?

There is no common measure of air quality and different proposals can be found in the literature. A frequently used approach is to assess air quality based on the average concentration of given pollutants at the time of the study. For example, Chay and Greenstone (2005) estimated air pollution based on the yearly average of county-level measures of TSPs. Similarly, Kim and Yoon (2019), Tang and Niemeier (2021), as well as Le Boennec and Salladarré (2017) assessed the air quality by using average values of pollutants from the studied period. Ou et al. (2022) presented some modifications of the above approach as the authors estimated the population-weighted annual means of PM_2.5_. Analysis of the mean concentration of a given pollutant in the real estate market context has some evident advantages. Some researchers (Renn 2008) have claimed that perception rather than facts largely determine human behaviour; hence, perceptible pollutants may influence the decisions of potential homebuyers more strongly. In general, people are more likely to experience air pollution when it can be sensed through visual and sensory feedback (Gatersleben and Uzzell 2000). The ozone or PM_10_, a visible component of smog, is likely to be noticed by potential home buyers and influence their decisions; thus, the inclusion of these aforementioned pollutants could be beneficial. However, this causes studies on the impact of “visible” and “invisible” pollutants are not fully comparable.

Another method to measure air quality and frequently cited in the literature is through a synthetic measure called an air pollution index. The construction of such a measure requires several steps. Firstly, one should estimate the weights of each pollutant contributing to the air quality, often with PCA (principal component analysis) or rotated PCA. PCA is a method for creating synthetic variables which contribute the majority of the information. Synthetic variables are linear combinations of original data, while the weighting scheme is the major output of PCA. If original variables are highly correlated or do not reveal variability, PCA may eliminate some of the variables and keep the data with unique and influential information. On the contrary, non-correlated variables are not reduced. Secondly, since there is a mismatch between the location of properties and measuring stations, one should interpolate the environmental variables. It is crucial first to interpolate single pollutants and then calculate a synthetic index of air quality because the variance of estimation error is smaller than when the order is reversed (Myers 1983). Many interpolation methods are available in geostatistics, but ordinary kriging appears to be the most reliable among others, including inverse distance weighting, splines, or Thiessen polygons (Anselin and Le Gallo 2006). When the weights are settled, and pollutants interpolated, one should calculate the air pollution index as the weighted average using the aforementioned estimates. The following approach can be found in Anselin and Lozano-Gracia (2008), Fernández-Avilés et al. 2012 and Lu and Lee (2022).

The conclusions from these studies are equivocal. Fernández-Avilés et al. 2012 suggest that the results inconsistent with hedonic theory might be due to objective pollution measures, such as API. This explanation is also in line with the presented theory emphasising the importance of perception in the decision process. Thus, it seems reasonable to include an air quality measure that can be directly sensed, e.g. through hearing or smell.

### The empirical study of air quality in Warsaw

According to the European Environment Agency (EEA), Polish cities are one of the most polluted. Based on the levels of fine particulate matter measured in over 300 European cities in 2019 and 2020, Warsaw (capital of Poland, main city of the Mazovian region) was ranked number 269 (EEA air quality viewer 2021). According to the Chief Inspectorate of Environmental Protection (in Polish: *Główny Inspektor Ochrony Środowiska*, GIOS), the principal sources of fine particulate matter pollution in the capital of Poland are anthropogenic emissions from the private residential sector and transport. Local authorities have made some efforts to mitigate the problem of domestic combustion of low-quality fuels, but the outcomes are far from satisfactory. Mazovia’s regional council adopted an anti-smog resolution to ban coal-fired individual household heating in Warsaw by 2023 (Warsaw 2017). Nevertheless, by 2022, there are still more ca. 6000 heating stoves (mostly private in individual households) to be removed (Warsaw 2022), and they disappear slowly (several hundreds of heating stoves per year), what enables achieving the objective. In 2019, the number of passenger cars per 1000 inhabitants in NUTS2 Warsaw region (*Warszawski Stołeczny*) was the eighth highest in Europe (EU regional average-540, Warsaw-729 cars per 1000 inhabitants) (EUROSTAT 2022a). According to a local authority report (Lee et al. 2022), Warsaw is witnessing a growing inflow of vehicles from people commuting to work from outside the city. Despite actions to improve air quality, there is still a need for policy enhancements.

The problem of air pollution has serious social consequences. Despite improvements across Europe, air pollution has continued to contribute to the severe burden of premature death and disease. In 2019, over 300,000 premature deaths of Europeans were associated with exposure to fine particulate matter. Air pollution leads to a wide range of diseases, including stroke, chronic obstructive pulmonary disease, cancers, aggravated asthma and lower respiratory infections. It also has a considerable economic impact, increasing medical costs and reducing life expectancy (European Environment Agency 2021). For instance, in 2018, the social cost per capita related to poor air quality in Warsaw was estimated at 2433 euros. The corresponding average cost for all cities included in the study was 1095 euros (Delft 2020).

For this study, pollution data were collected from the commercial Airly service (https://airly.org/pl/), the company that maintains sensors assessing air pollution in cities worldwide. The data were for 125 different locations in Warsaw City (the area of the city is 517 km^2^, 1.8 million inhabitants) and included daily (24-h average) measurements from December 2020 to January 2022 for particulate matters of three sizes (PM_10_, PM_2.5_, PM_1_), temperature, wind speed and wind direction — in total 40,214 measurements.

The data-cleaning procedure was based on verifying outliers with another source of information. Untypical data from 125 Airly sensors were cross-checked with data from GIOS. This governmental institution responsible for environmental protection initiatives has six measurement stations in Warsaw, and their historical data are publicly available. Potential outliers (high values) from Airly data that significantly differed from nearby observations from GIOS stations were removed. However, we decided to keep even relatively high measurements occurring during the day with GIOS alerts concerning poor air quality, assuming that the concentration of pollutants was extremely high during a given day. Cases with highly probable errors in number notation were eliminated. Data from monitoring stations were benchmarked with European Union standards. The acceptable daily average concentrations of PM_10_ and PM_2.5_ are 50 µg/m^3^ and 25 µg/m^3^, respectively. The corresponding annual norms are 40 µg/m^3^ for PM_10_ and 20 µg/m^3^ for PM_2.5_. There are no standards (neither by the European Union nor by WHO) for PM_1_.

**Fig. 1:**
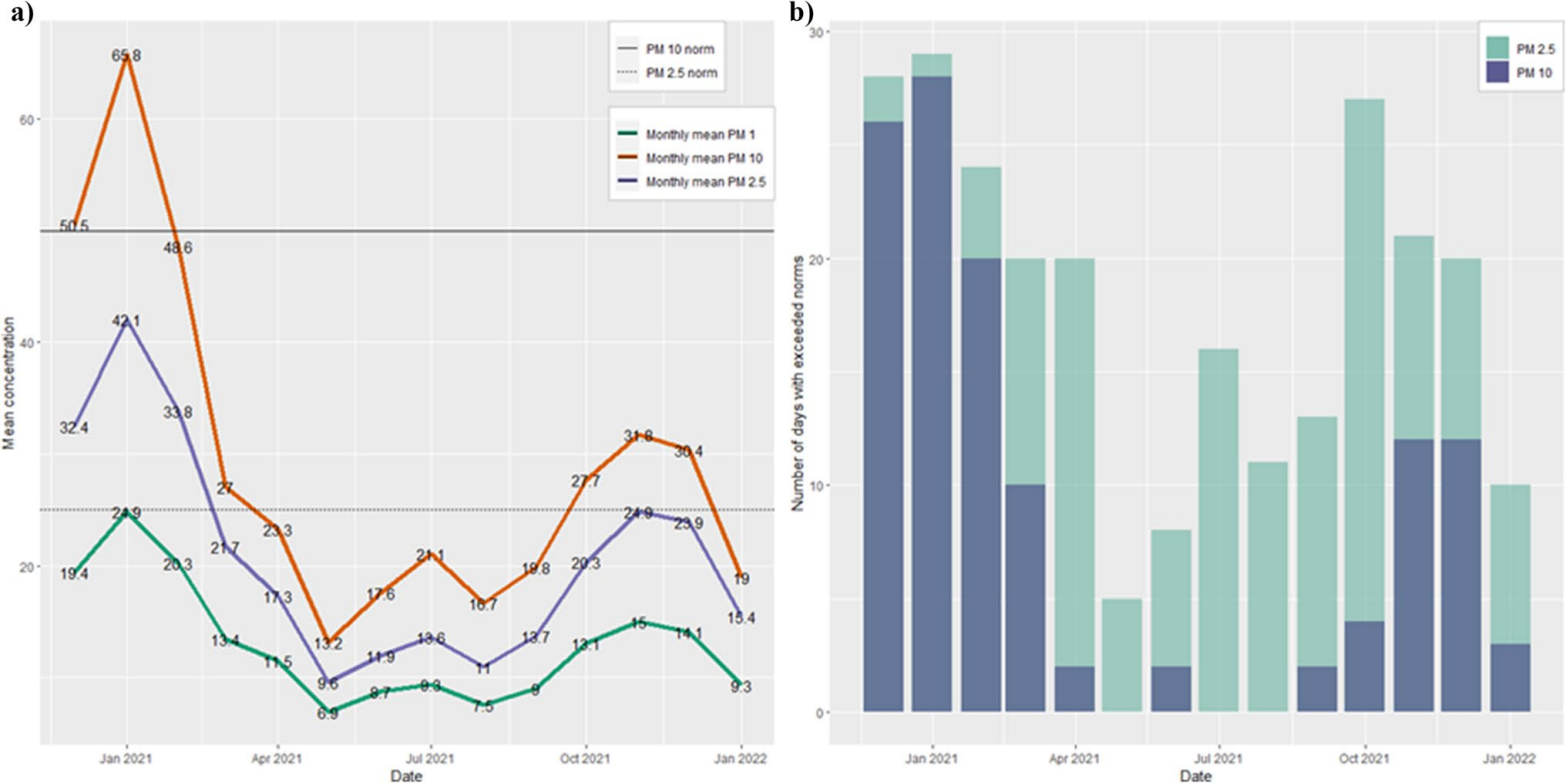
Air pollution in Warsaw: **a** monthly mean concentration of particulate matters from December 2020 to January 2022; **b** number of days per month with exceeded norms of PM_10_ and PM_2.5_. Source: Own work based on Airly data and ggplot2 R package

In the analysed period of 14 months, the highest monthly mean concentration of any particulate matter occurred during the winter months, especially from December 2020 to February 2021 (Fig. 1a). This phenomenon, in some part, can be confirmed by GIOS alerts occurring relatively frequently during this period. The primary explanation for this poor air quality is meteorological conditions impeding pollutant spread in a situation of increased emissions related to increased demand for heating and heavy traffic in the city centre. The best air quality in terms of the mean concentration of particulate matter and the number of days without exceeding European standards was registered during the summer. Comparing the concentration of particulate matter with European standards (Fig. 1 b) brings the sad conclusion that inhabitants experienced poor air quality during the majority of days in the winter. The trend in the number of days with exceeded PM_2.5_ standards was markedly different from that for larger diameter particles. Unlike PM_10_, ambient fine particulate matter of PM_2.5_ norms were surpassed throughout the year. One of the main sources of PM_2.5_ pollution is fuel combustion which includes residential energy use, car exhaust and industrial processes (McDuffie et al. 2021). Therefore, referring to the GIOS report, which states that the leading causes of air pollution in Warsaw are transport and domestic heating, the obtained results are, to some extent, explainable by those factors.

According to the GIOS’s report (GIOS 2021) on annual air quality assessment, meteorological factors such as wind speed and direction, temperature or precipitation determine the spread of pollutants. In the GIOS’s alerts, a commonplace explanation for poor air quality was weather conditions preventing the spread of pollutants. Thus, below, we verify if atmospheric factors significantly influence poor air conditions in winter. We consider two significant elements of the weather: wind and temperature.

**Fig. 2:**
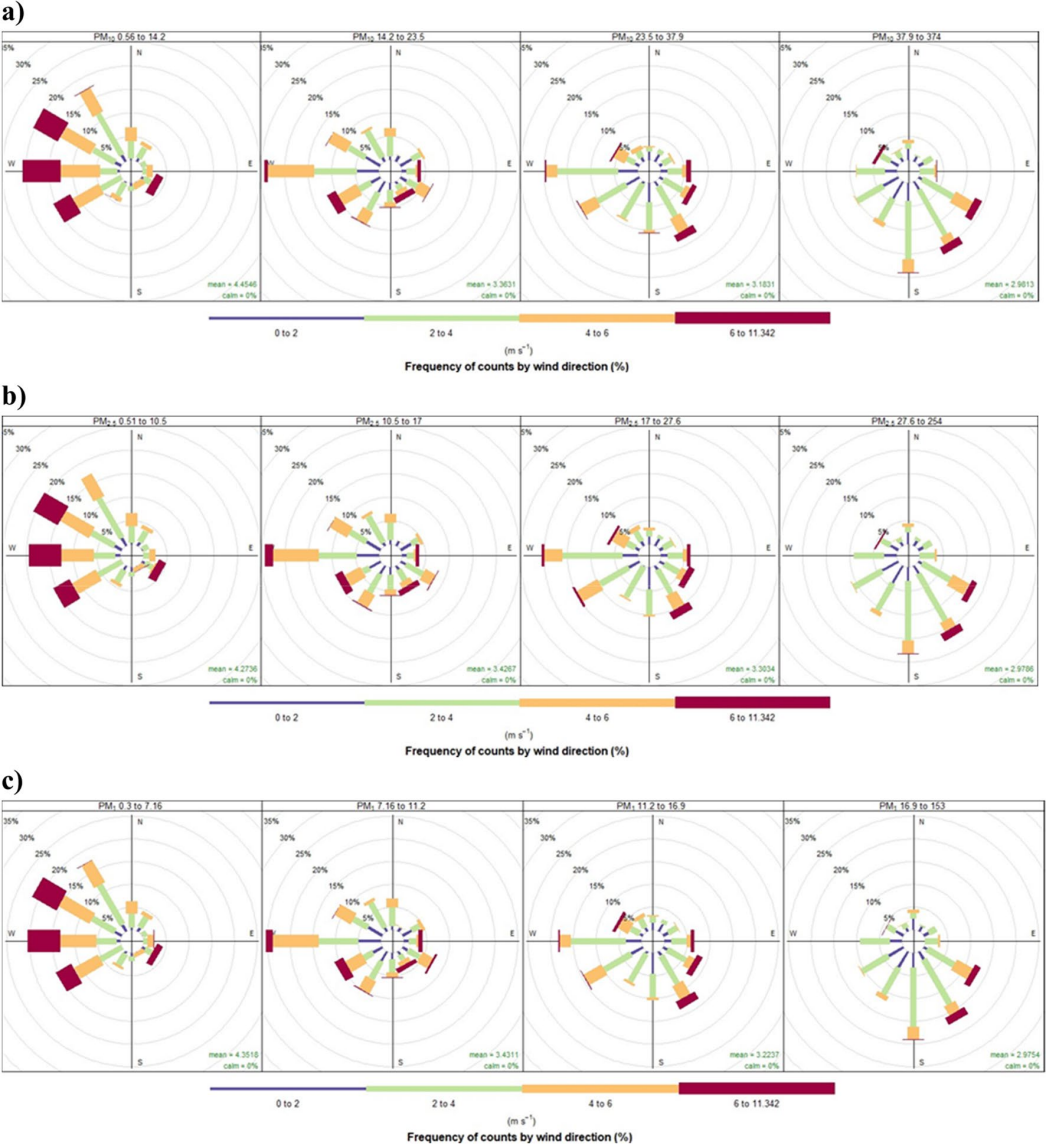
Pollution rose for 2021 by pollutants: **a** PM_10_; **b** PM_2.5_; **c** PM_1_ — each panel for a different level of PM. Source: Own work based on Airly data and openair R package

Wind analysis is based on wind roses (Fig. 2) splitting the pollutant concentration for each quantile level. Different width “paddles” represent the wind speeds. Besides data concerning wind, they are based on pollutant concentrations from 2021 split into four quantiles. The plots show a proportion, represented as a percentage of time that the wind is from a certain angle and the wind speed range when pollution values are within a given interval. The lowest concentration of every type of particulate matter is associated with the wind dominated by a westerly direction with relatively high speeds. In contrast, the highest concentrations are correlated with low-speed winds from southern directions. In the Appendix, one can find wind roses by months from December 2020 to January 2022 and spatial distribution of sensors with weather and pollution values for a selected date. Analysing all data regardless of time, it turns out that southern and southerly-east winds contribute most to overall high concentrations, while easterly winds contribute to relatively good air quality. A comparison of pollutant roses with graphs showing wind speed and direction in a given month confirms that during the worst months in terms of air quality, the weather conditions were frequently unfavourable to the spread of pollutants.

**Fig. 3:**
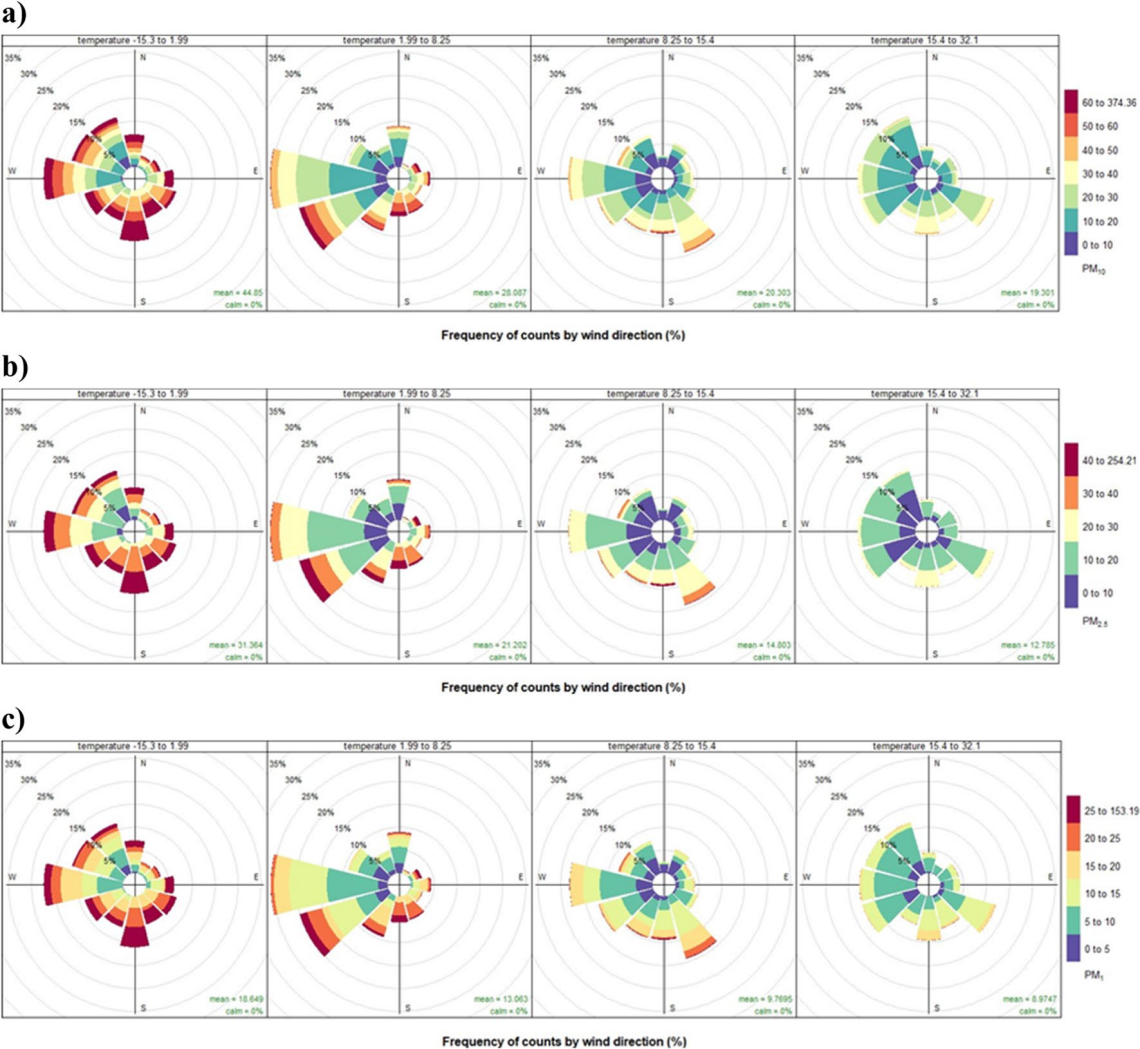
Pollution rose by pollutants and temperature: **a** PM_10_; **b** PM_2.5_; **c** PM_1_ — each panel for a different level of temperature. Source: Own work based on Airly data and openair R package

When investigating the temperature impact in parallel with the wind direction on the air quality (Fig. 3), typically a lower temperature leads to a higher pollutant concentration. The interpretation of the aforementioned graphs is similar to the wind roses. For each pollutant, the temperature measurements were divided into four quantiles. Then, together with information on wind direction, one can observe which wind direction and temperature contribute most to the overall concentration of particulate matter. For instance, only west and north-west winds favour relatively good air quality during the coldest period of the year. On the contrary, the lowest concentration of pollutants is associated with the highest temperature and westerly winds. All things considered, our analysis proves that, to some extent, the poor air quality visible in the data can be explained by the atmospheric conditions.

**Fig. 4:**
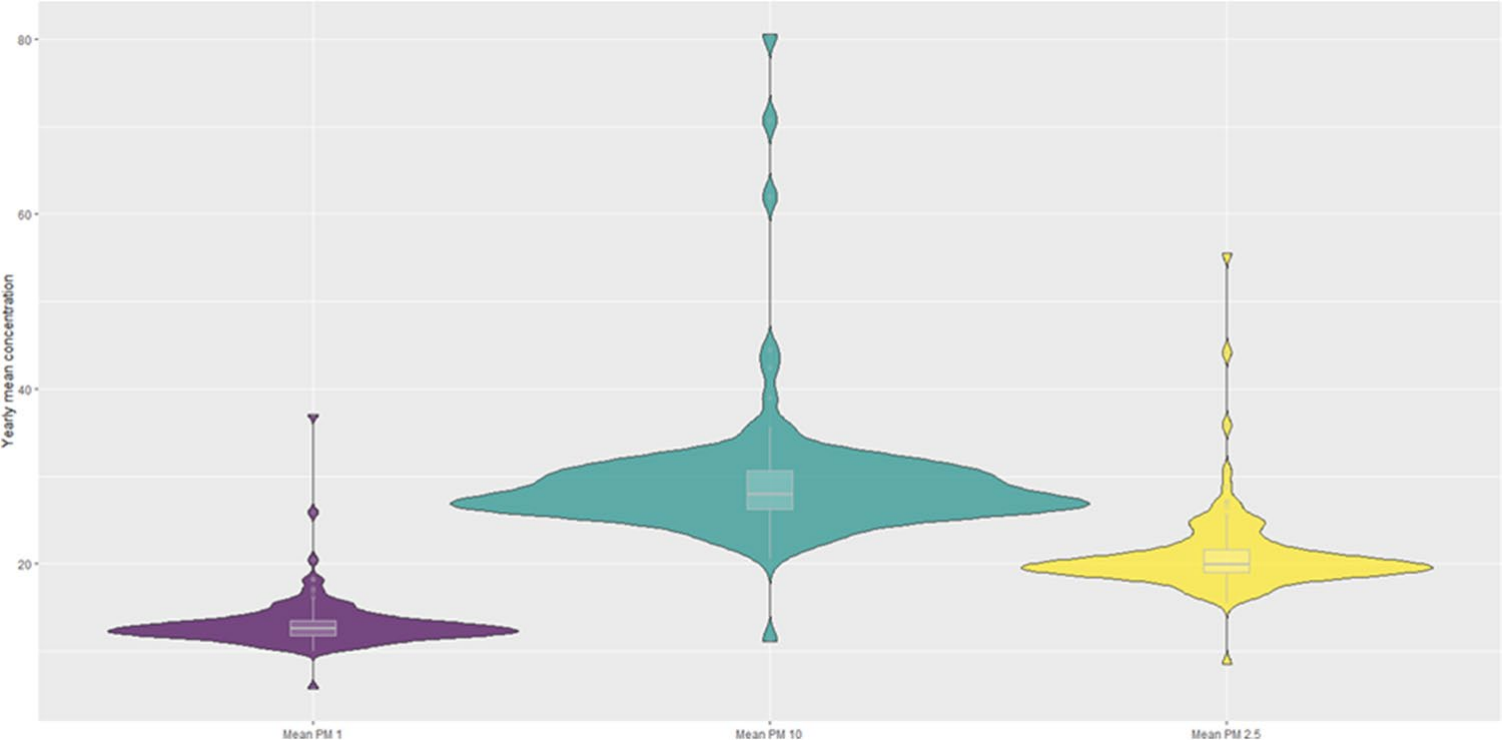
Violin plot of the yearly mean concentration of PM_10_, PM_2.5_ and PM_1_. Source: Own work based on Airly data and ggplot2 R package

To investigate the variability in the data on annual mean dust concentrations between different locations, we created a violin plot (Fig. 4). The charts enable a comparison of the distribution of pollutants’ measurements. Overall shape and distribution of the PMs are similar for all types of contamination with a very low number of outliers. The broader regions of the plot indicate values that occur more frequently. In this case, most observations are concentrated around the median, indicating very low data variability. In other words, the spatial variability of the mean concentration of particulate matter across the city is low as the average measurements for different locations are very similar to each other.

#### Reflections on air quality measures

Considering the air quality, an important issue is how to assess it. Some studies (e.g. Ou et al. 2022; Chay and Greenstone 2005) treat cities or counties as a single territory by giving the unique value of pollution and neglecting internal diversity. However, major cities are large territories (e.g. Prague 496 km^2^, Warsaw 517 km^2^, Berlin 892 km^2^, Rome 1285 km^2^, London 1572 km^2^); thus, local variation may matter and more detailed spatial information is needed. Values of pollution can be linked to housing transactions using k nearest neighbours in case of densely located measurement stations e.g. this study, 125 sensors for 517 km^2^ (one sensor per 4.1 km^2^) or apply statistical methods as Thiessen polygons, inverse distance weighting or kriging to get approximation of surface in case of sparsely located pollution sensors, e.g. Anselin and Le Gallo (2006) using 27 sensors for 1738,7 km^2^ (one sensor per 64.4 km^2^). When considering point data, it is important to know how they are used to create a synthetic air quality measure. The most common approaches in the literature are to use a weighted average of the concentrations of the analysed particles or to include in the model the mean concentration of each pollutant. In the first case of weighted average, creating an air quality index requires weights, often based on (rotated) PCA (Anselin and Lozano-Gracia 2008; Fernández-Avilés et al. 2012; Lu and Lee 2022). The details of the above method are described in the literature review.

Another approach is to use the air quality measure developed by public institutions dealing with environmental issues. The commonly used measure of air quality in the European Union is the European Air Quality Index (EAQI, https://www.eea.europa.eu/ themes/air/air-quality-index). The index is based on the concentration values for up to five pollutants, including PM_10_, PM_2.5_, ozone, nitrogen dioxide and sulphur dioxide. According to the European Environmental Agency, “it reflects the potential impact of air quality on health, driven by the pollutant for which concentrations are poorest due to associated health impacts”. However, this method has some drawbacks. Firstly, to take full advantage of the benefits of this approach, it is necessary to have data concerning ozone, nitrogen dioxide and sulphur dioxide. Not all of the substances mentioned above may be reported in full coverage (e.g. for Warsaw, nitrogen dioxide and ozone are reported from four stations, and sulphur from one station only, while PMs are reported from 125 locations). Obtaining spatially dense data (e.g. in a grid, as a surface, or a neighbourhood of points) may require interpolating some measurements using some interpolation techniques, such as kriging, to calculate the EAQI. However, in Warsaw’s case, there is a considerable imbalance between the number of stations measuring concentrations of particulate matter and other pollutants. The results of the obtained index could be biased. Omitting some of the variables is also not a solution. Each type of pollution has a different health impact. Additionally, as explained in the literature review, perceptible pollutants strongly impact consumer decisions. For example, the ozone, one of the most visible pollutants in the form of “smog” (Fernández-Avilés et al. 2012), may remarkably influence potential homebuyers’ perception of air quality in a given location; thus, its omission is not recommended.

The composites of the index are also under question. By relying on EAQI, one neglects the PM_1_ data, as it is not included in this index. This is probably due to the majority of medical science literature widely proving the adverse effects of PM_10_ and PM_2.5_ on human health, while exposure to PM_1_ is still under investigation. However, some papers confirm positive associations between PM_1_ and total and cause-specific respiratory diseases (Hu et al. 2022). More studies are needed to assess quantitatively and qualitatively the effects of exposure to PM_1_ on human health. From a statistical perspective, the inclusion of PM_1_ reduced the risk of omitted variables.

We tried the aforementioned approaches in an empirical analysis of pollution in Warsaw. First, we used rotated PCA to calculate the weights required for the weighted average of our data. However, the obtained loading values (weights) were very similar; therefore, the index would be the standard arithmetic mean, and no information would be reduced. In addition, the cumulative variance explained by the data concerning air quality was very low (20%); thus, this approach was not continued. Secondly, we tried reconstructing the EAQI at the local level. In this case, the poor availability of data for ozone, nitrogen dioxide and sulphur dioxide made it impossible. Moreover, we could not have used the PM_1_ data due to a lack of standards for this pollutant in aggregated air quality measures. All of the factors described above contributed to the decision to include the annual average concentration of PMs in the models. To check the robustness of our approach while modelling, we estimated models including the arithmetic mean of pollutants as an environmental variable. As the weights from unsatisfactory PCA were almost equal to one for each pollutant, we decided to use a simple arithmetic mean. However, models with the mean concentration of each pollutant outperformed this approach; therefore in the latter analysis, we use the aforementioned method.

### Analysis of housing market data in Warsaw

Data for housing prices were collected following the approach of Efthymiou and Antoniou (2013) with a Python web scrapper from Poland’s largest real estate website, otodom.pl.^2^ Data for sale offers of apartments in Warsaw were collected between 20 December 2021 and 15 February 2022 with a weekly web scraping frequency. After removing property offers without address or total price, with improbable characteristics or duplicates, the size of the final data set is 15,000 observations (see Appendix for spatial distribution of offers). Offer data often arouses the discussion, favouring transactional data. However, as Lyons (2013) showed using a data set concerning Ireland’s real estate market from the period 2001–2012, asking prices and transaction prices are closely correlated in space and time, without visible bias due to location. Therefore, in the absence of final transaction information, offer prices may be perceived as a good approximation of transaction prices, as they are linearly rescaled.

From a theoretical point of view, it is worth considering the choice of sales transaction prices instead of rents. During the last 12 years, over 80% of Poland’s population lived in a household which owned their home (EUROSTAT 2021). When comparing the Polish rental market with other European Union countries, it seems that it is of inferior quality and less institutionalised, probably due to its small size. Hence, as Poles prefer to own a property rather than rent one, it seems reasonable to expect a more accurate reflection of their preferences in sales prices (ING 2019).

According to Picard et al. (2010), a home is a bundle of four types of characteristics: physical attributes of the property, characteristics of the surrounding neighbourhood such as demographics, accessibility (to green areas, central business district, etc.) and environmental quality. Along with the house attributes parsed directly from the real estate website, three additional variables were created. Using the {gmapsdistance} R package and Google Cloud Platform, the transit time from the given location to the city centre was estimated. The chosen date for the calculation was Tuesday, 22 March 2022, at 08:00 AM. The purpose of the described variable was to reflect a time when people would likely be commuting by public transport from their apartment to the metro station Centrum, the city centre and transport hub. Moreover, the distance to the city centre was also calculated. Applying proper functions from {sf} and {osmdata} R packages to the property address enabled the creation of dummy variables indicating to which district a given address belongs. Over a dozen house attributes were parsed; however, only those with less than one-fifth of missing data were considered in further analysis. Consequently, deleted variables such as monthly cost of maintenance, construction material or type of windows were not taken into account.

The dataset was divided into two submarkets: the standard market, with a total apartment price below 1.5 million PLN (ca. 333,000 EUR), and the premium (exclusive) market, with a total price above 1.5 million PLN. Threshold amounts result from the average salary in Warsaw.^3^ We expect that investors’ preferences in both markets differ. In line with that, the summary statistics (Tables 1 and 2) presented below show the main characteristics of two groups of properties — standard and premium.^4^

**Table 1:** Summary statistics — standard submarket

Statistic	Q1	Mean	Median	Q3
Price per sqm (PLN)	10,432.6	12,845.3	12,383.6	14,498.8
Total price (PLN)	514,000	691,129	632,071	810,000
Surface area (sqm)	41.5	55.7	52.4	65.7
Year of construction	1975	1997.7	2008	2021
Number of rooms	2	2.6	2	3
Floor	1	2.8	2	4
Floors in building	3	6.1	5	8
Market type (secondary)	0	0.7	1	1
Market type (primary)	0	0.3	0	1
Air conditioning	0	0.1	0	0
Garage	0	0.5	1	1
Elevator	0	0.6	1	1
Balcony	0	0.6	1	1
Garden	0	0.1	0	0
Terrace	0	0.1	0	0
Mean PM_10_	26.2	32.4	28.6	31.2
Mean PM_2.5_	18.9	22.9	20.3	22.7
Mean PM_1_	12.1	14.4	12.9	14.2
Distance to centre (km)	3.9	6.4	6.1	9
Transit time (min)	22.6	29.8	29.7	35.9

Source: own calculations based on housing and Airly data

**Table 2:** Summary statistics — premium submarket

Statistic	Q1	Mean	Median	Q3
Price per square metre (PLN)	16,102.2	20,760.6	18,838.3	24,000
Total price (PLN)	1,800,000	2,818,438	2,268,000	2,990,000
Surface area (sqm)	97	137.6	126	159.8
Year of construction	2002	2001.1	2012	2020
Number of rooms	3	4	4	5
Floor	1	3.1	3	4
Floors in building	3	5.3	4	7
Market type (secondary)	0	0.7	1	1
Market type (primary)	0	0.3	0	1
Air conditioning	0	0.2	0	0
Garage	0	0.7	1	1
Elevator	0	0.7	1	1
Balcony	0	0.6	1	1
Garden	0	0.1	0	0
Terrace	0	0.3	0	1
Mean PM10	26.9	32.4	29.3	31.2
Mean PM2.5	18.9	22.5	20.2	23.5
Mean PM_1_	12.1	14	12.9	13.7
Distance to city centre (km)	1.8	4.5	4.3	6.1
Transit time (min)	16.2	23.8	23.2	31.3

Source: own calculations based on housing and Airly

Core variable — air quality — required a few aggregation decisions that were taken based on deep analytics. We have started with individual data — for each day, for each particulate matter of three sizes (PM_10_, PM_2.5_, PM_1_) and for each of 125 stations. We had to deal with space, time and composition. In the spatial dimension, for each sale offer, we assigned one sensor location using the criterion of nearest neighbour and data from this sensor were linked to real estate features. In the temporal dimension, we considered a few strategies: from day-by-data to one-observation data. The most efficient option (in terms of computation and information included) was to derive the annual average of particulate matter. In the cross-sectional dimension, we also considered a few strategies (discussed previously) — the most efficient one was to include each pollutant separately; therefore, model includes mean values of PM_10_, PM_2.5_ and PM_1._

**Fig. 5:**
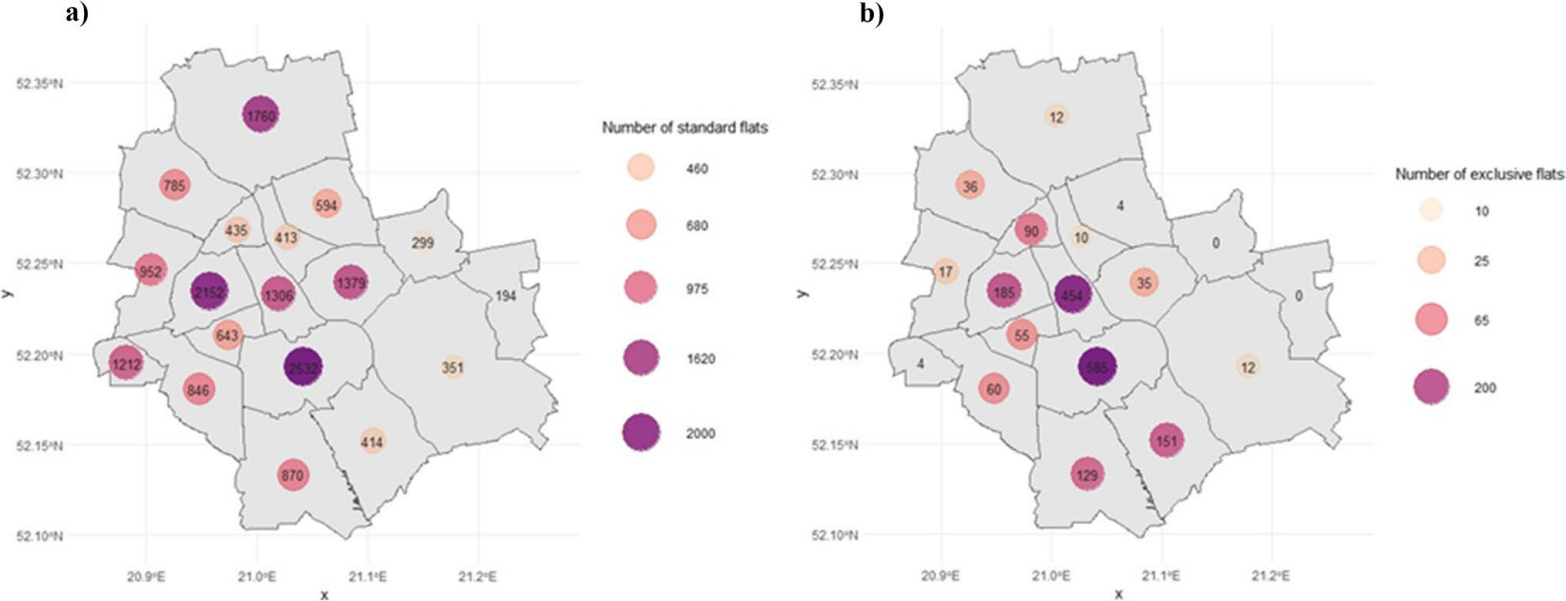
Spatial distribution of sales offers in Warsaw in 2022: **a** standard submarket, **b** premium submarket. Source: Own work using data from otodom.pl, with the use of {sf} and {osmdata} R packages

The domain of the study includes all 18 districts of Warsaw — the capital and largest city in Poland. Figure 5 presents the geographic variation in a number of properties over city districts, divided into submarkets (standard or premium). One should underline that Warsaw is free of heavy industry and the main sources of pollution are from the residential sector and transport.

### The hedonic model with air quality — methodology and results

Early real estate modelling research used OLS, sometimes with spatial fixed effects (like census tract fixed effects) which is a way of controlling for spatial relations. However, in the case of spatial geo-localised point data, its geographic dimension as neighbourhood and relative location should be included as an additional variable (Anselin and Bera 1998). To examine the existence of spatial autocorrelation, global Moran’s *I* statistic was estimated using the k-nearest neighbours (*knn*) spatial weights matrix W. The hypothesis of spatial randomness can be rejected and one observes significant links between prices in a given location and its neighbourhood (Moran’s *I*_standard_ = 0.569, Moran’s *I*_premium_ = 0.538, both *p*-value < 0.001), which justifies spatial modelling (Kopczewska 2020).

Initially, we screened seven spatial hedonic models (GNS, SAC, SDM, SDEM, SAR, SEM, SLX)^5^ to estimate the influence of air quality on housing prices. Subsequently, model evaluation based on the number of significant variables, no-opposite effect of *rho*, *lambda*, *theta* and possibly the lowest AIC (Akaike Information Criterion) revealed that SEM (spatial error model) outperformed the other models for both standard and exclusive submarkets. In our research, the only environmental variables included are mean concentrations of air pollutants. Therefore, the inclusion of the spatial lag of the error term was crucial as it incorporates the effect of omitted environmental variables such as noise, green areas in the vicinity or distance to roads. As other models with λ had higher AIC or a smaller number of significant variables, the SEM model turned out to be the best option. It assumes the unobservable correlation of neighbours’ features and includes the spatially autocorrelated error terms as follows:

$$\begin{array}{c}Y=\beta_0+X\cdot\beta+u\\u=\lambda\cdot W\cdot u+e\end{array}$$

where *Y* is a vector of flat prices per square metre (dependent variable), β_0_ is a constant term, β represents coefficients for a set of explanatory variables *X* of a given property (including air quality data) and *u* is an error term, which includes λ·*W*·*u* — the spatial lag of error term (based on spatial weights matrix W) and standardised error term *e*. The autoregressive factor λ·*W*·*u* of error *u* is interpreted as the average error of neighbouring locations; thus, it enables the modelling of unobservable or difficult-to-measure features (Kopczewska 2020).

**Fig. 6:**
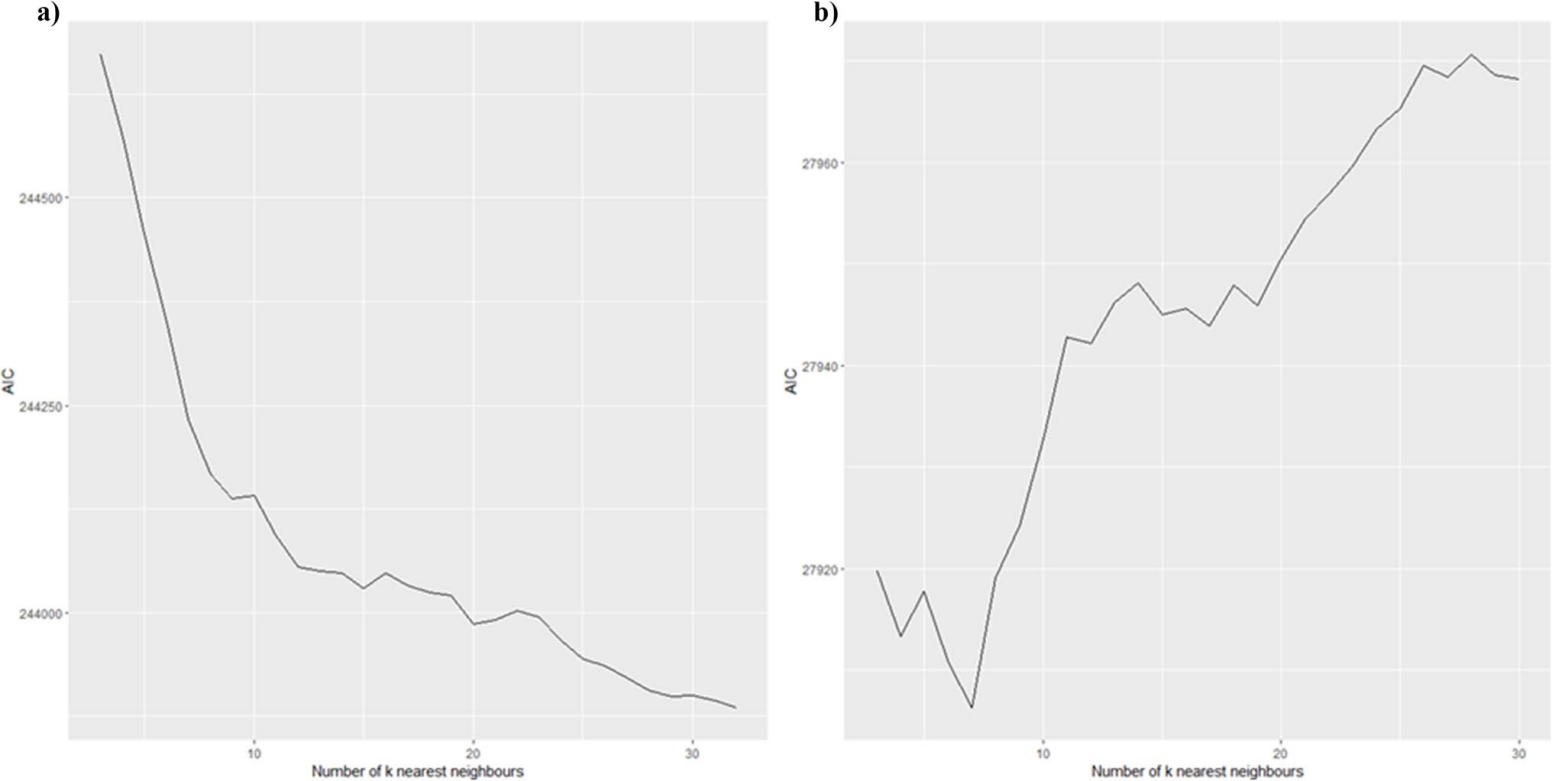
Optimisation of AIC by selecting k-nearest neighbours *knn* for spatial weights matrix W: **a** standard submarket; **b** exclusive submarket. Source: Own work with the use of {ggplot2} R package

When using spatial econometrics one should consider the neighbourhood structure as it influences the modelling of locational dependencies and interactions. The spatial weights matrix W constitutes the basis of spatial analysis as its fundamental role is to capture the relations between neighbours and translate them into a form utilisable for statistical procedures (Fingleton and Arbia 2008; Bavaud 1998; Griffith et al. 2018). In real estate modelling on point data one has limited choice of W — mostly one selects the k-nearest neighbour matrix. However, one must decide on the number of neighbours to incorporate into the matrix. Too small *k* may underestimate the strength of spatial dependencies, while including too many *k* may lead to bias in spatial parameters (Kubara and Kopczewska 2023). When searching for optimal *k* in W, one should select the model with the lowest AIC (Burnham and Anderson 2004). The basis underlying the concept of *k* optimisation is that inaccurate W tends to result in wrongly specified parameters, which in turn more strongly affects the AIC value than in the most optimal scenario (Kubara and Kopczewska 2023). To determine the most accurate value of the discussed parameter, we compared the AIC of SEM models (separately for both submarkets) incorporating spatial weights matrix W with the number of neighbours between *knn* = 3 and *knn* = 30. As shown in Fig. 6, in the case of the models based on the luxury apartments data, the AIC function reaches its minimum for *knn* = 7. At the same time, no clear minimum of the function was found for the standard flat models. Thus, assuming that the optimal parameter value should not be too big, *knn* = 9 was considered in further modelling of properties with lower prices. Table 3 summarises the estimation results for SEM models with W *knn* = 9. Secondly, we controlled for interactions; however, they were not significant and they reduced the magnitude of the model estimates.

**Table 3:** Spatial error models for submarkets

Dependent variable: price of a square meter of a flat		Standard flats	Exclusive flats
		(1)	(2)
Internal features of the flat	Surface area (sqm)	− 36.59***	10.36***
	Year of construction	29.02***	36.24***
	Number of rooms	− 270.41***	− 858.58***
	Floor	21.19**	− 99.00
	Floors in building	− 49.85***	− 78.01
	Market type (secondary)	1120.63***	1533.71***
	Air conditioning	709.32***	949.89**
	Garage	477.42***	− 181.82
	Elevator	310.65***	638.83*
	Balcony	− 221.80***	− 363.79
	Garden	45.04	755.41
	Terrace	99.67	777.06**
Districts of Warsaw	Bialoleka	− 943.59***	861.48
	Bielany	735.38***	5973.90**
	Mokotow	1193.84***	2934.15
	Ochota	515.90**	− 123.84
	Praga Polnoc	7.53	939.74
	Praga Poludnie	− 289.42	3864.10
	Rembertow	− 1547.48***	2496.67
	Srodmiescie	3224.36***	6497.90***
	Targowek	− 817.08***	− 1879.58
	Ursus	− 914.99***	3565.19
	Ursynow	1651.40***	5955.07**
	Wawer	95.25	7658.26**
	Wesola	1205.13***	3483.14
	Wilanow	2876.65***	6459.78***
	Wlochy	− 455.91**	1701.08
	Wola	1162.64***	2233.34
	Zoliborz	2216.74***	3782.91*
Relative location	Distance to city centre	− 366.55***	− 1641.29***
	Transit time	− 14.63**	99.83**
Air quality	Mean PM_10_	− 60.07***	− 89.05
	Mean PM_2.5_	92.58**	− 421.30
	Mean PM_1_	− 12.31	811.13**
	Constant	− 41,112.88***	− 49,346.83***
	λ	0.485***	0.382***
	Observations	13,539	1399
	Log Likelihood	− 122,031.70	− 13,918.14
	_σ_2	3,815,348.00	24,991,628.00
	Akaike Inf. Crit	244,137.50	27,906.28
	Wald test (df = 1)	1939.54***	111.83***
	LR test (df = 1)	1545.56***	92.71***

*p < 0.1; **p < 0.05; ***p < 0.01

The specification of models involved variables expected to impact the price per square metre (sqm) of the property. The results are divided into three categories: structural, location and environmental variables.

*The structural variables* include all property features. The coefficients of the year of construction, market type, air conditioning and the number of rooms are significant. Their signs correspond to intuitive expectations. The transaction sqm price for standard flats decreases by almost 37 PLN (0.3%) with an additional sqm; as in Warsaw, the smallest flats are the most expensive per sqm. In the case of exclusive properties, the surface area surprisingly turned out to have an inverse effect. This observation might be counter-intuitive as the conclusions in the literature indicate the positive influence of the surface area (Kim and Yoon 2019; Tang and Niemeier 2021). However, in most studies cited, the authors created one model including all observations regardless of their total price. In addition, as shown in Table 2, luxury apartments are, on average, much larger than standard ones; therefore, those of greater size might be perceived as even more prestigious when compared to smaller flats.

Individuals seeking to purchase a regular property prefer those located on the top floors of low-rise buildings. The price per sqm increases by 21.19 PLN (0.16%) with each stock and declines by almost 50 PLN (0.4%) as the number of floors in the building grows. Such an effect is not observed in the exclusive flats model as both variables are insignificant. Considering outdoor living spaces, a terrace generates an additional value of approximately 777 PLN (3.7%) per sqm of the exclusive apartment, while the balcony in a regular apartment reduces the sqm price by almost 222 PLN (1.7%). As the former seems reasonable, the latter is a bit striking. The access to a garage positively influences the price per sqm of standard apartments, as parking on the street is challenging due to a limited number of free parking places.

*The location category* includes mainly dummy variables indicating Warsaw’s districts of the property location. Appendix Fig. 9  presents a map of Warsaw districts. Additionally, transit time by public transport and distance to the city centre are considered in both models. For regular apartments, the location in Mokotów, Ochota, Wola or Żoliborz increased sqm prices by 4–17%. Those districts are adjacent to the central part of the city and have relatively good access to public transport (e.g. with a metro line). The peripheral district Wesoła, relatively recently incorporated into Warsaw’s administrative boundaries, still has a significant share of green spaces in its total surface area, which increases the prices of standard real estate (9.4%). A considerable part of the housing infrastructure there constitutes estates of single-family houses, which, jointly with the green surroundings, may attract homebuyers. Regular apartment locations in Targówek, Ursus, Włochy, Białołęka and Rembertów districts negatively influence property prices (by 3–12%) as all mentioned regions are peripheral. Moreover, the first three areas have an industrial character that may reduce real estate value. In line with expectations and the literature (Le Boennec and Salladarré 2017), for both types of dwellings, central locations in the Śródmieście district increased their value (25% in the standard submarket and 31% in the premium submarket). Also, standard as well as exclusive apartments situated in Wilanów, Bielany or Ursynów, due to their locations, were at a premium compared to other city areas (5–25% in the standard submarket and 28–31% in the premium submarket). There is no surprise in the positive sign of the coefficient indicating whether the given flat is in Wilanów, as it has always been a district which is famous for luxury real estate. In the Ursynów area, the potential explanation for the positive influence on price is the metro line. As one could expect, properties situated closer to the city centre have a higher sqm price than those further apart, regardless of their total value. This relation is reflected by the significant and positive value of the coefficient specifying distance to the city centre; nevertheless, the effect is more visible for exclusive apartments, which is probably due to their concentration in central areas.

Lastly, the impact of transit time by public transport varies between standard and luxury flats. The former benefits from a shorter commuting time, while the effect is the opposite considering the latter. Little is known about the impact of real transit time using public transport. Most of the papers examine the influence of transportation infrastructure in the neighbourhood on real estate value, which may not fully reflect the real possibilities of using public transport. However, in our case, the opposite signs in the two models might be explained by homebuyers’ needs. While individuals with lower budgets seeking to purchase a flat may use public transport on an everyday basis, people who can afford luxury apartments may ignore this factor.

*The environmental variables* include the yearly mean concentration of particular matters: PM_10_, PM_2.5_ and PM_1_. The statistically significant coefficients of PM_10_ and PM_2.5_ in the model related to standard apartments indicate that their prices are associated with air pollution levels. However, while PM_10_ negatively impacts the house value (by 0.5%), according to our estimates, PM_2.5_ increases the sqm price (by 0.7%). Similar conclusions can be drawn for PM_1_ in the case of exclusive apartments (by 3.9%). The insignificant coefficients of PM_10_ and PM_2.5_ for luxury properties, as well as the estimates of PM_1_ for standard properties, evidence a lack of association between air pollution and housing prices. The results may be unexpected; however, there is some speculation about why this occurs. Fernández-Avilés et al. 2012 and Tang and Niemeier (2021), using different pollutants and techniques to measure air quality, have drawn similar conclusions on the influence of air pollution on real estate value. Among studies which have published insignificant results relating to air pollution impacting house prices, some authors have acknowledged that the irrelevant effect of environmental coefficients is because the given pollutant does not tend to exceed air quality standards (Kim et al. 2003). Examining the literature, the results of other research suggest that when, in general, ambient air pollution is relatively low, the pollutants’ influence on property value is more likely to be insignificant. However, compared to other metropolitan cities in Europe, air quality in Warsaw is quite poor. Moreover, as explained in the section concerning air quality data analysis, during winter in the worst scenario, the number of days with exceeded norms of PM_10_ and PM_2.5_ exceeded the level of 20. Altogether, neither the insignificant coefficient of the PM_1_ in the model describing standard properties nor estimates of PM_10_ and PM_2.5_ for luxury flats are, as suggested by other researchers, are related to superior air quality. The significant and negative impact of PM_10_ on the prices of standard flats, as suggested by Lu and Lee (2022), may be explained by the fact that particulate matter with the largest diameters is more perceptible than other pollutants included in the study below. The positive sign displayed by the PM_2.5_ coefficient in the standard real estate model, as well as PM_1_ increasing the value of exclusive properties, constitute counter-intuitive results. According to Fernández-Avilés et al. 2012, these unexpected conclusions may arise from the difference between the subjective environmental perception of homebuyers and objective conditions. The rational consumer makes the decision which maximises utility based on all available information. However, the environmental condition of a given property may be out of the scope of a potential homebuyer. Unlike long-term air pollution data, information on the accessibility of public services or transportation is readily available. Therefore, in the Polish context of high demand for real estate, purchasing a house might not depend on the air quality in the area but on affordability and facilities available in the vicinity. Based on the analysis of the spatial distribution of PM_10_, PM_2.5_ and PM_1_, it happens to be that, in most cases, the most polluted districts have the most developed commercial services, communication and leisure facilities, etc. For instance, Bielany and Śródmiescie in both models positively impact the sqm price of a dwelling. The former is the central district of Warsaw, while the latter is a former industrial area with a steel mill, which is today an attractive place to live. As the conclusions from the literature about the impact of air quality on housing prices are ambiguous, it seems that in general, nations around the globe differ in their attitude toward clean air value. In a significant number of the papers based on data from Asia, air pollution decreases real estate prices, while outcomes of studies referring to other parts of the world are not as straightforward, which is also the case in Warsaw.

As one can observe, according to *p*-values, not all variables in both models are significant. The main purpose of the research was to question whether air quality impacts real estate prices; thus, its foremost role is explanatory. As written by Shmueli (2010), “trimming potentially theoretically meaningful variables is not advisable unless one is quite certain that the coefficient for the variable is near zero, that the variable is inconsequential, and that trimming will not introduce misspecification error “. Hence, some insignificant variables, important from the theoretical point of view, are included in the final models.

### Policy implications

The analysis of air quality data has highlighted the need for improvements in Warsaw’s air pollution policy as the ambient air quality over the analysed time was unsatisfactory. Compared to other European cities, the capital of Poland experiences some of the most severe air pollution, mostly from fuel combustion from the residential sector and transport, which leads to comparatively high social costs per capita. Therefore, municipal actions towards those two sources of pollution to improve air quality should be reconsidered.

In the case of pollution from transportation, one of the most common approaches widely applied worldwide is the restriction of access to the city centre for the most polluting cars. According to the European Cyclist Federation (ECF, 2020), more than 250 low-emission zones (LEZ) are established in the European Union. Such a strategy was implemented in Madrid, Spain. In 2018 local authorities established Madrid Central (MC), a low-emission zone intended for air pollution reduction and the promotion of new mobility behaviours. Lebrusán and Toutouh (2021) considered the NO_2_ concentration from December 2013 to November 2019 (5 years before the implementation of MC and 1 year after). The authors compared the NO_2_ concentration itself, average differences in measurements before and after MC, and the percentage of time when the population was exposed to levels which met the European standards of NO_2_. Their results indicate a significant difference in mean NO_2_ concentration between the period before MC and after. Moreover, the authors proved that after establishing the low-emission zone, the population was exposed to good-quality air for more time. The positive effect of the implementation of MC has been noticed not only in the central area but in the whole city. However, one should note that although there was a reduction in the NO_2_ concentration in winter, there was no statistically significant change between the time before and after establishing the low emission zone. The overall positive effect of MC was also confirmed by Lebrusán and Toutouh (2021) who proved that the concentration of NO_2_ decreased by more than one-third. Therefore, in Madrid’s case, the implementation of pedestrianisation brought substantial benefits.

Another study examining the effectiveness of low-emission zones was conducted in Berlin and Munich, Germany (Gu et al. 2022). The effects of LEZs on PM_10_ and NO_2_ were evaluated using the general additive mixed model. The influence of car restrictions in both cities was estimated by considering the air pollution concentration, including measurements from the regional background and seasonality effects (public holidays, season, wind direction, etc.). Overall, the results indicated that low emission zones effectively reduced PM_10_ concentrations, while the results for NO_2_ were equivocal. Gehrsitz (2017) examined the impact of low-emission zones, introduced at different points in time in different cities throughout Germany, indicating the reduction in air pollution in terms of PM_10_. The results of the study demonstrated that the implementation of the stage 1 zone (a ban for only the dirtiest vehicles) was associated with a decrease in PM_10_ daily mean concentration by 1.5–2.5%. In comparison, more strict restrictions allowed for a reduction of 3 to 4%. There is no low emission zone in Warsaw; however, local authorities plan to establish one by 2023–2024. Based on the results of LEZs from the cited literature, the introduction of car restrictions in the city centre appears to be a promising initiative to improve air quality in the capital of Poland.

In the case of pollution from the residential sector, according to the European Commission (EC 2022), heating and cooling constitute about half of the EU’s energy consumption. In 2020, only 23% of the total energy used in this sector was from renewable sources (EUROSTAT 2022b). As the achievement of European climate targets cannot be accomplished without any change in the cooling and heating sector, a variety of legislations and tools have been implemented to support the development of renewable energy use (e.g. 2016 EU Heating and Cooling Strategy, 2018 Renewable Energy Directive, European Green Deal). Household heating is one of the major contributors to air pollution in Warsaw. The ambient air quality in the city is poor, and municipal policies, especially the efficiency of the stove removal program, have been unsatisfactory. Therefore, to meet the objectives of European directives apart from the improvement of citizens’ wellbeing, reconsideration of current actions is needed. In reducing air pollution associated with domestic heating, one should note that buildings’ energy efficiency is also an essential factor. In this case, local authorities are relatively active as a citizens’ assembly on energy efficiency has taken place and ended with binding recommendations (*Warszawski Panel Klimatyczny*, Warsaw Climate Panel). Aside from this, Warsaw has established several other initiatives, such as standards for green buildings (Warsaw 2022) or the SONNET project (Sonnet 2022), aimed at researching social innovations that accelerate the transition from fossil fuels. Most of the mentioned programs are under development; thus, their assessment is impossible.

Results from our model bring ambiguous conclusions and show some irrationality in potential homebuyers’ decisions. Those conclusions may be related to the lack of information on long-term environmental conditions in a given district. Investors would probably consider this factor if the information on general air quality was easily available in addition to temporal measurements of air pollution. Overall, such consciousness could be beneficial for local authorities. In particular, air pollution–related deaths could decrease. In the context of air quality assessment, the authors believe that local authorities should invest in monitoring stations. Only four stations report nitrogen dioxide and ozone, while the measurements of sulphur are available from just one location. Warsaw is a metropolitan city with a relatively large surface area compared to other European cities. Thus, drawing valuable conclusions from the available information is very difficult. Also, the estimation of EAQI seems worthless as obtained conclusions do not capture the complete picture of the actual air quality in Warsaw. The European measure for air pollution is based on numerous health studies; hence, its implementation in the analysis could shed light on the examined matter.

## Conclusions

This paper has investigated the ambient air quality impact on the real estate market in Warsaw, the capital city of Poland. Based on detailed transaction data, web scraped from the most popular real estate website combined with precise air quality information from over a hundred monitoring stations in Warsaw, we have provided an insight into the capitalisation of environmental amenities in house values.

As many other researchers (e.g. Anselin and Le Gallo 2006), we underline that the way the pollution data are included in hedonic models for real estate matters for the outcome. Therefore, it is crucial to understand the nature of those data, especially in spatial and temporal dimensions. We found out that spatial variability of the average concentration of particulate matter across the city is low — different locations are very similar to each other in terms of average values of pollutants in the air. On the other hand, the temporal variability of pollution data is very high. This is because a major source of pollution in Warsaw is transport and coal-based heating in private houses. First, the emissions of those pollutants are highly seasonal and weather-dependent. Heating and individual transportation intensify in winter due to low temperatures. Secondly, the spillover and persistence of pollutants in the air also depend on the weather — strong westerly winds improve the air quality (as they blow out the particulates outside the city), while low-speed winds from southern directions tend to worsen contaminant levels. Based on statistical inspection, this study proposed the form of using pollution data — yearly mean concentrations of individual particulate matter. This method was applied as no other existing methodologies in the literature on air quality measurement gave satisfactory results: neither the air pollution index understood as the weighted average of pollutants concentrations with the weights derived from PCA, nor the European Air Quality Index, which does not include the concentration of PM_1_. Estimated PCA weights were equal, which does not reduce any dimension, while the impact of PM_1_ should not be ignored as current research results indicate adverse health outcomes (Hu et al. 2022). Additionally, as the air quality together with atmospheric conditions changes throughout the year, it is crucial to include in the research a reliable measure of air pollution, not biased by seasonal anomalies. Together with the role of perception in consumer decisions, the mean concentration of pollutants, especially those that can be seen or smelled, seems to be the most suitable way for incorporating environmental variables into economic research.

The second part of the study linked air pollution with housing valuation. The novelty proposed in this paper was to estimate separate models for two submarkets: standard properties and exclusive flats, as homebuyers seeking to purchase a luxury property may have different preferences than people interested in homes of lower values. The models used spatial econometrics, unlike the non-spatial hedonic approach insensitive to spatial dependencies. We used spatial error models (SEMs), incorporating k-nearest neighbour spatial weights matrix W optimised in terms of AIC. However, the results indicated insignificant or counter-intuitive outcomes for air quality. The empirical findings of this research mostly do not confirm the negative impact of poor air quality on real estate value (only PM_10_ slightly impacted the housing prices in the standard market). Thus, the results do not support the hedonic theory.

There are a few potential explanations for this phenomenon. The first explanation relates to the different nature of housing markets and the spatial allocation of attractive real estate. Most studies proving the inverse effect of air quality on real estate were conducted in the USA or Asia, which may simply differ from the European market. Different investors’ preferences may matter, and it is highly probable that housing prices are primarily associated with services, availability of transport, economic activity in the neighbourhood, etc., but not with environmental amenities — especially in Europe. Secondly, in the empirical literature, one can find that when overall air quality is relatively good, potential homebuyers are not willing to pay for the improvement in environmental conditions. However, in the case of Warsaw, a capital that faces poor air quality compared to other European cities, it seems highly improbable. Customers who have got used to poor air quality may not be aware of the high value of non-polluted air. Thirdly, investors may not be rational due to a lack of available information related to air quality. While the general up-to-date information on observed pollution in the city is commonly available (through mobile apps, websites and press), the historical data broken down into districts or neighbourhoods are not easily available. Thus, when the environmental factor is not fully known, it cannot be adequately valued, which is reflected in insignificant air quality coefficients. The fourth explanation lies in the methodology for the inclusion of air quality data. Our study tested all known approaches, but we believe that deeper studies on how to include environmental data are needed. We see potential opportunities in using machine learning solutions to enhance the database quality and development of air quality assessment methods.

## Figures and Tables

**Fig. 7:**
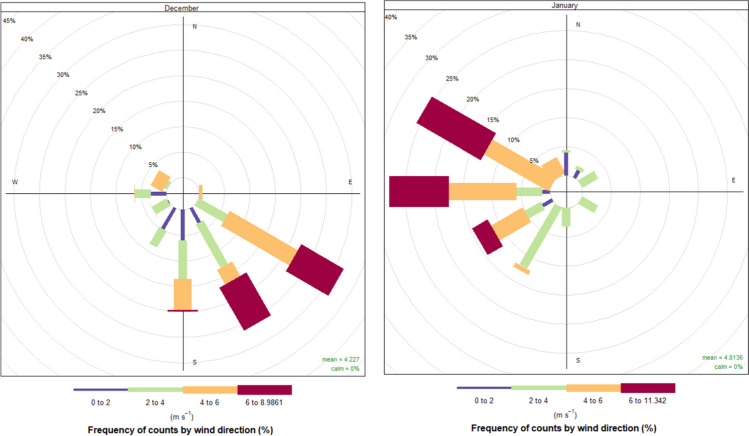
Wind roses for December 2020 and January 2022. Source: Own work based on Airly data and {openair} package in R

**Fig. 8:**
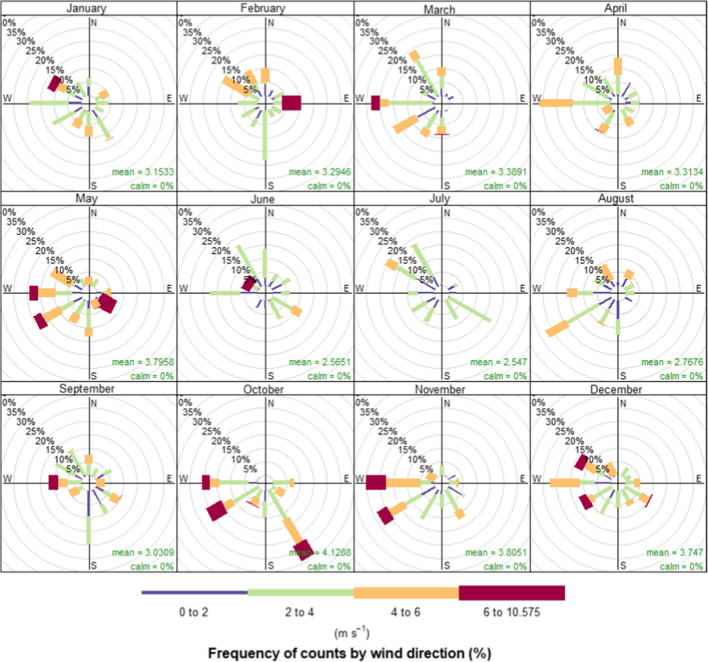
Wind roses for 2021 by months. Source: Own work based on Airly data and {openair} package in R

**Fig. 9:**
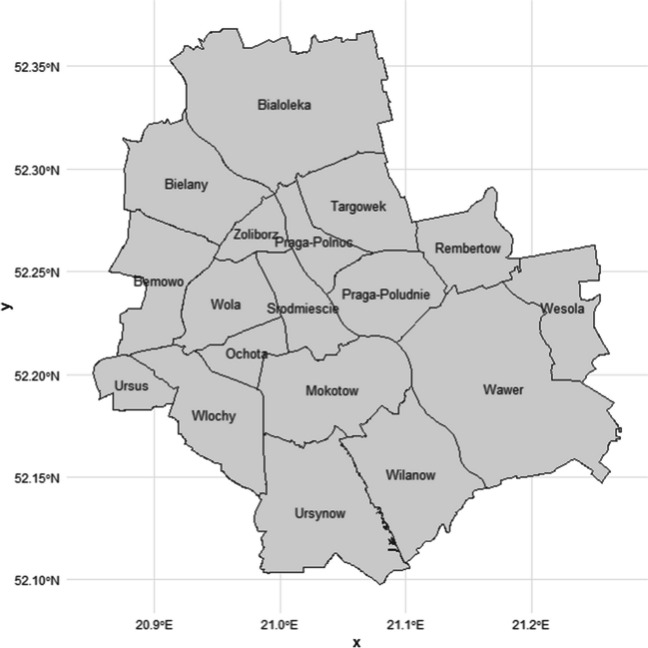
Map of Warsaw districts. Source: Own work with the use of {osmdata} and {ggplot2} R packages

**Fig. 10:**
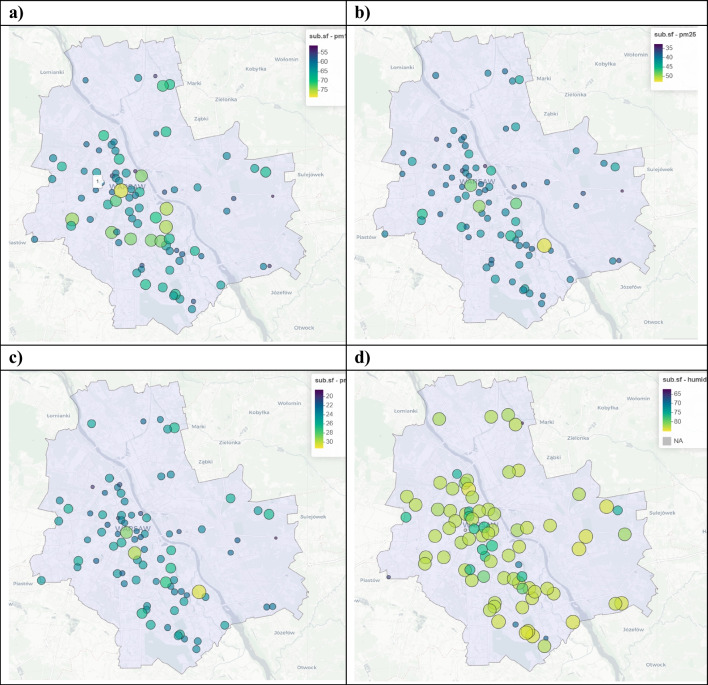
Daily weather and pollution of particulate matter: a) PM_1_, b) PM_2.5_, c) PM_10_, d) humidity (on 1st December 2020). Source: Own work based on Airly data and {sf} and {mapview} packages in R

**Fig. 11:**
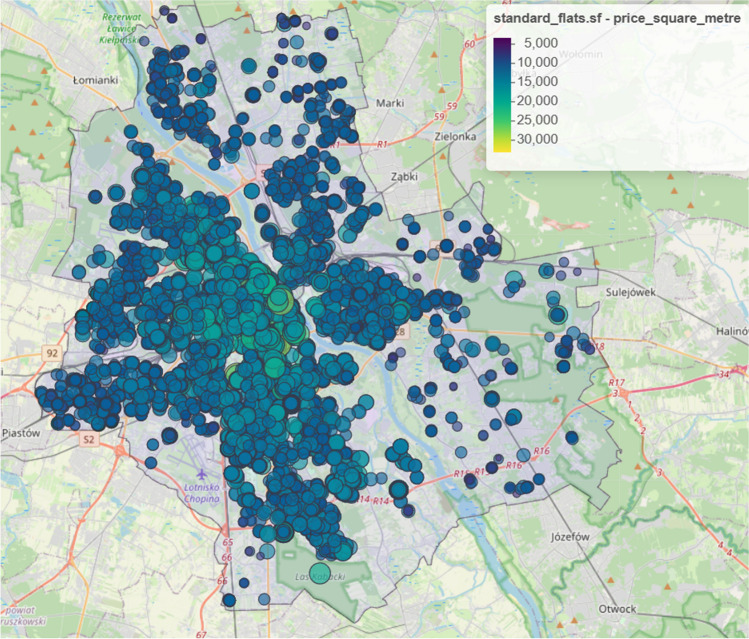
Prices (per sqm) of standard flats. Source: Own work based on web-scraped data and {sf} and {mapview} packages in R

**Fig. 12:**
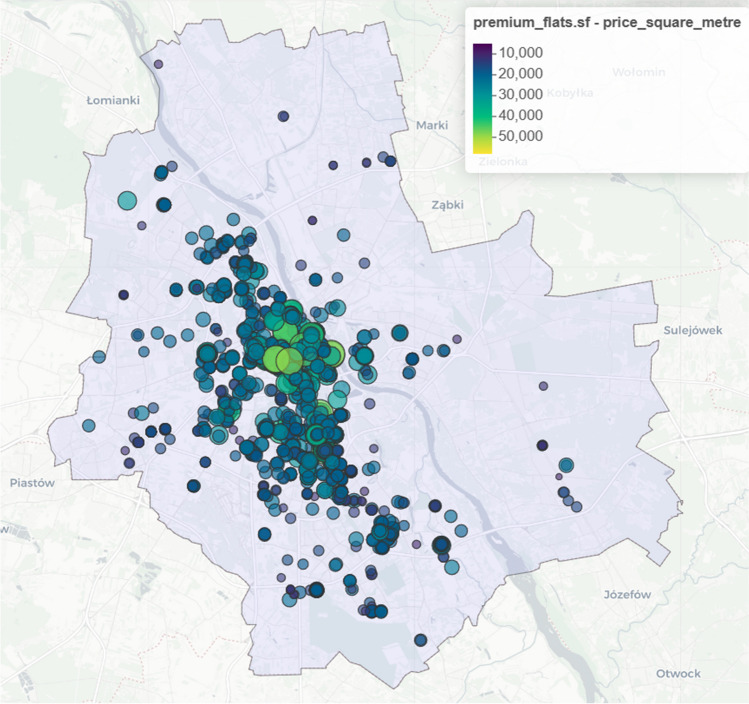
Prices (per sqm) of premium flats. Source: Own work based on web-scraped data and {sf} and {mapview} packages in R

## Data Availability

The data used in this study cannot be shared for legal reasons.

## Appendix

This appendix includes additional visualisation of data used in the study. In Fig. 7, one can find wind roses for each month from December 2020 to January 2022. Figure 8 summarises monthly data by direction and by different wind speed categories. Wind speeds, split into intervals, are represented by different width “paddles”. The grey circles show the percentage frequencies. The plots show the proportion, expressed as the percentage of time wind that is from a certain angle and speed range. More examples and interpretations of wind and pollution roses can be found in: https://bookdown.org/david_carslaw/openair/wind-roses.html (Access: July 2022). Figure 9 presents the division of Warsaw into districts. Figure 10 presents the location of pollution and weather sensors and the values for selected day. Figures 11 and 12 present the prices (per sqm) of analysed housing transactions on standard and premium markets.

## Footnotes

1 In our research, we use the word *investors* to refer to people willing to buy a property for their own use as well as developers building houses for sale. The two groups share the same goal — to maximise their utility/profits; therefore, clean air in the neighbourhood is advantageous for both. In the case of individuals, the theory explaining this approach is presented in the literature review.

2 In the Polish context, detailed real estate data sets are usually unavailable from authorities or housing websites. Online platforms do not provide API for data collection. According to Boeing and Waddell (2017), commercially maintained data sources and information provided by local authorities represent a limited sample of households; thus, they can produce inaccurate estimations and conclusions. To effectively gain insight into the real estate market, one should consider using nontraditional data sources.

3 The average salary in Warsaw in 2022 is ca. 8500 PLN gross, which makes ca. 5600 PLN net. The majority of inhabitants can afford apartments for which the loan instalment does not exceed 60% of single net income, ca. 3400 PLN, which is for a loan of 800,000 PLN for 30 years at APR 3%. With some personal contribution to the investment, the cost of a flat approaches 1,500,000 PLN as a maximum.

4 Data are consistent with international statistics for 1 m^2^ of flat in the city centre (https://www.numbeo.com/cost-of-living/city_price_rankings?itemId=100). Warsaw listed there with price USD 4500 per m^2^ is a bit cheaper than Manchester and Liverpool but more expensive than Bologna, Doha, Brussels, Chicago, Saint Petersburg, Dubai, etc.

5 Spatial models may include the spatial lag of *y* (rho, *ρ*), spatial lags of *X* (theta, *θ*) and the spatial error (lambda, *λ*) and, following Elhorst (2010), can be classified as follows: full model (GNS, Manski) with *ρθλ*, SAC with *ρλ*, SDM with *ρθ*, SDEM with *θλ*, SAR with *ρ*, SEM with *λ* and SLX with *θ*.
